# A systematic review and meta-analysis of *GFAP* gene variants in Alexander disease

**DOI:** 10.1038/s41598-024-75383-4

**Published:** 2024-10-17

**Authors:** Alice Grossi, Francesca Rosamilia, Silvia Carestiato, Ettore Salsano, Isabella Ceccherini, Tiziana Bachetti

**Affiliations:** 1 Laboratory of Genetics and Genomics of Rare Diseases, IRCCS Istituto Giannina Gaslini, Genoa, 16147 Italy; 2 Clinical Bioinformatics, IRCCS Istituto Giannina Gaslini, Genoa, 16147 Italy; 3 Department of Neurosciences, Rita Levi Montalcini University of Turin, Turin, 10126 Italy; 4 SC Malattie Neurologiche Rare, Fondazione IRCCS Istituto Neurologico C. Besta, Milano, Italy; 5 IRCCS Ospedale Policlinico San Martino, Genova, Italy; 6 UOSD Laboratory of Genetics and Genomics of rare Diseases, IRCCS Istituto Giannina gaslini, Via G Gaslini, 5, Genova, 16148 Italy

**Keywords:** Alexander Disease, GFAP, Meta-analysis, Genotype-phenotype correlation, Variant effect, Diseases, Neurological disorders, Neurodegenerative diseases, Genetics, Genotype

## Abstract

Alexander disease (ALXDRD) is a rare neurodegenerative disorder of astrocytes resulting from pathogenic variants in the *GFAP* gene. The genotype-phenotype correlation remains elusive due to the variable expressivity of clinical manifestations. In an attempt to clarify the effects of *GFAP* variants in ALXDRD, numerous studies were collected and analyzed. In particular, we systematically searched for *GFAP* variants associated with ALXDRD and collected information on the location within the gene and protein, prediction of deleteriousness/pathogenicity, occurrence, sex and country of origin of patients, DNA source, genetic testing, and clinical signs. To identify possible associations, statistical analyses and meta-analyses were applied, thus revealing a higher than expected percentage of adult patients with ALXDRD. Furthermore, substitution of Arginine, the most frequently altered residue among the 550 predominantly missense causative *GFAP* variants collected, were mostly *de novo* and more prevalent in early-onset forms of ALXDRD. The effect of defective splicing in modifying the impact of *GFAP* variants on the age of onset of ALXDRD was also postulated after evaluating the distribution of the corresponding deleterious predictive values. In conclusion, not only previously unrecognized genotype-phenotype correlations were revealed in ALXDRD, but also subtle mechanisms could explain the variable manifestations of the ALXDRD clinical phenotype.

**Supplementary Information:**

The online version contains supplementary material available at 10.1038/s41598-024-75383-4.

## Introduction

Alexander disease (ALXDRD; OMIM#203450) is a rare disorder caused by variants in the gene encoding the Glial Fibrillary Acidic Protein (*GFAP*) (HGNC:4235). With the exception of one participant known to date, all *GFAP* variants are heterozygous, thus conferring the disease autosomal dominant inheritance. ALXDRD is a leukodystrophy belonging to the astrogliopaties since the primary damage occurs in astrocytes where the GFAP protein forms cell-specific type III cytoskeletal intermediate filaments.

*GFAP* alpha is the main isoform, including 432 amino acids encoded by 9 exons. The resulting protein is composed of a central “rod domain”, characterized by alpha-helical secondary structures and divided into four regions 1A, 1B, 2A, and 2B, encoded from exon 2 to exon 6, and two non-alpha helical domains at the N-terminus and C-terminus of the GFAP protein, encoded by exon 1 and exons 7–9, respectively. In addition, other regions not organized into secondary structures are represented by L1 (between 1A and 1B), L1-2 (between 1B and 2A) and L2 (between 2A and 2B).

ALXDRD symptoms are not homogenous among patients and criteria have been proposed to classify patients manifesting different forms of the disease. Patients can be distinguished based on the age of onset (AOO) of the disease: infantile (0–2 years), juvenile (3–12 years), and adult (> 13 years), characterized by progressively increasing severity^1^. A further classification in the infantile subgroup has been introduced to recognize neonatal ALXDRD, i.e. infants aged less than 30 days of age, who occasionally present with hydrocephalus secondary to aqueductal stenosis^2^. While infantile patients are generally characterized by seizures, megalencephaly, gradual loss of intellectual function, and developmental delay, adults are affected with bulbar/pseudobulbar, pyramidal tract, and cerebellar signs in addition to dysautonomia and occasional gait disturbance and ocular abnormalities^1^.

More recently, radiological signs have been used to classify ALXDRD patients into Type I and Type II^3^. Type I manifests before the age of 4 and is characterized by classic radiological signs (frontal predominance of white disturbances; periventricular rim of low signal on T2/high signal on T1) while type II can manifest throughout life and presents atypical radiological signs such as atrophy of the brainstem and cerebellum and posterior predominance. In both classifications, the intermediate and mixed forms are indicated as juvenile^3^ and type III^4^, respectively.

Since the discovery of *GFAP* as the causative gene of ALXDRD, the pathogenetic mechanisms induced by *GFAP* variants have been investigated in vitro^5,6^ and in vivo^7,8^. Both cellular and animal models expressing mutant GFAP showed cytoplasmic aggregates identical to the Rosenthal fibers (RFs) found in patients’ astrocytes^9^, containing intermediate filaments, such as GFAP, vimentin, and synemin, in addition to members of the protein quality control, such as the small heat shock proteins (sHSPs) alphaB-crystallin, and HSP27, plectin, and the cell cycle protein Cyclin D2. In RFs, both wild-type and mutant GFAP are trapped, thus suggesting that *GFAP* variants exert a dominant negative effect, consistent with the “sequestration hypothesis”^10^.

Furthermore, the expression levels of GFAP appear to modulate the pathogenesis of ALXDRD, likely acting on the penetrance and expressivity of the variants^11^ and opening new perspectives on the down-regulation of GFAP gene expression as a therapeutic target^12^.

So far, only a few articles have reported large collections of *GFAP* variants. In addition to the very early reports^3,13–15^, some more recent manuscripts have focused on specific subgroups of patients such as the infantile form^16,17^ or the late-onset forms^18^.

In this work, we present a comprehensive review of all *GFAP* variants identified in ALXDRD starting from the discovery of the causative gene until the end of 2023. For each variant we collected information on the location within the gene and protein, prediction of deleteriousness/pathogenicity, occurrence, sex and country of origin of patients, source of DNA, genetic testing and clinical signs. In addition, we have performed several statistical analyses and meta-analysis to investigate possible yet unknown associations.

## Results

As of 12/29/2023, a total of 1048 papers were identified by searching the Pubmed (https://pubmed.ncbi.nlm.nih.gov/*)*, Web of Science (https://www.webofscience.com/wos*)* and Waisman Center (https://alexander-disease.waisman.wisc.edu/*).* Thirty additional relevant studies were manually retrieved after the database search. After removing 475 duplicates and 15 untraceable papers, 588 unique publications underwent independent screening of title and abstract by 2 reviewers (A.G. and F.R.). A total of 402 publications were excluded due to one of the following reasons: not Alexander disease, non-human disease, commentary or review reporting no new cases, experimental details only, full text not English or inaccessible, inconclusive genetic data and/or reported incorrectly.

**Fig. 1:**
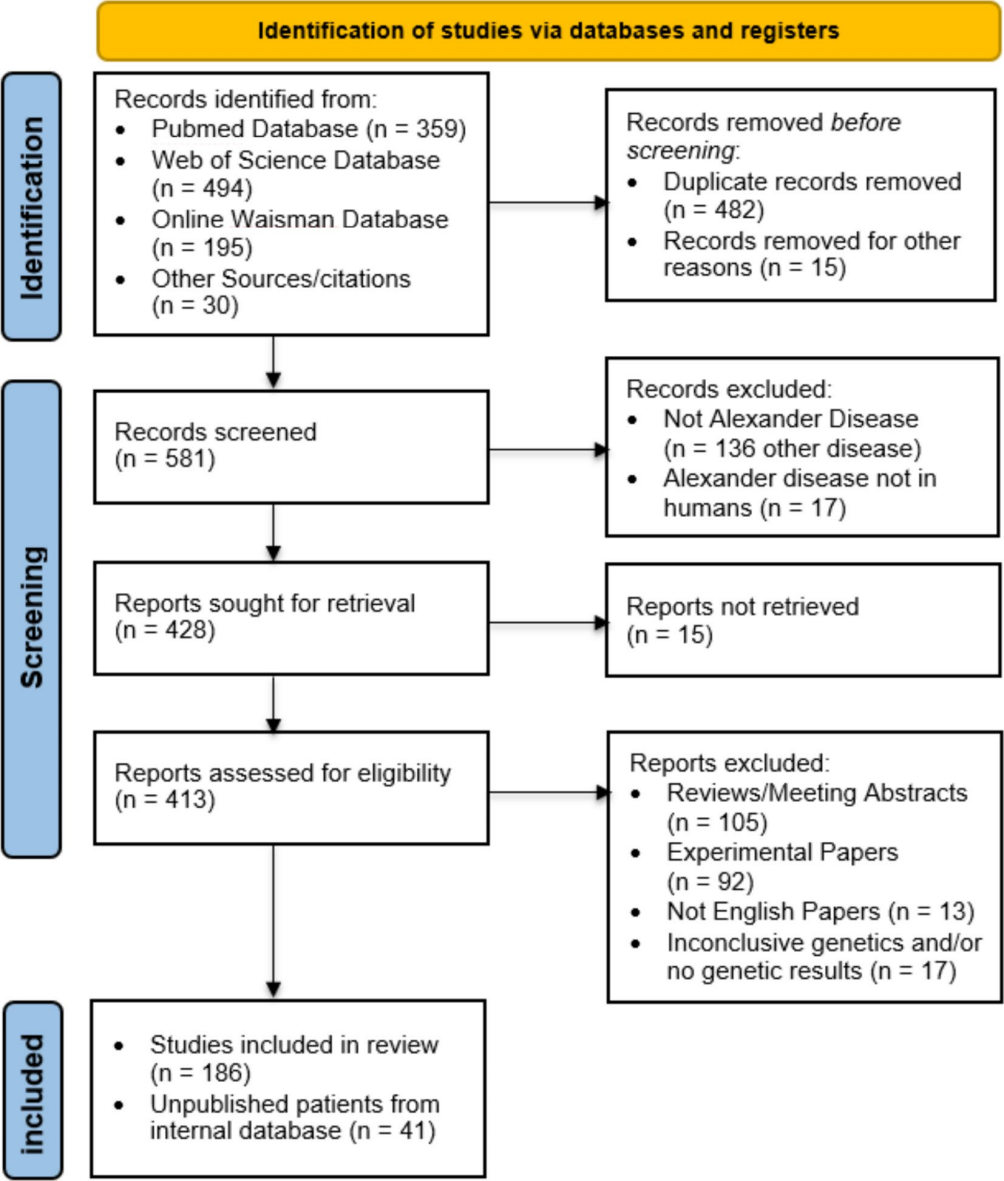
PRISMA workflow. The PRISMA workflow outlines the systematic approach for conducting a meta-analysis. The diagram provides a visual representation of the paper’s selection process and outlines the study identification, screening, eligibility, and inclusion steps.

Overall, 186 papers with at least one new ALXDRD case and with clinical and genetic details were suitable for inclusion in this study (Supplementary Table S1). The selection process is detailed in Fig. 1. For each manuscript, the geographical location of the first author’s research group was collected. Grouping by continent, the geographical distribution of contributions on ALXDRD is as follows: 89 papers from Asia (in particular, 59 from Japan), 59 from Europe (of which 19 from Italy), 38 from North and South America, 4 from Oceania, and 0 from Africa.

### *GFAP* gene variants retrieved

From the 186 papers, we identified a total of 550 ALXDRD participants carrying 182 different causative variants in the *GFAP* gene, as reported in Supplementary Table S2.

Subsequently, we analysed each variant relatively to the GFAP transcript ENST00000253408.11, evaluating the HGVS nomenclature for both protein and cDNA changes, the specific protein subdomains affected, and the variants’ proximity to exon-intron junctions.

Additionally, we evaluated the potential impact of each pathogenic variant using the CADD scoring system as a measure of deleteriousness, the ACMG classification as a pathogenicity score, and classified effects on protein structure or function in terms of missense, nonsense, splice-site, frameshift, deletions or synonymous changes. For all variants, the specific amino acid alterations were noted. We also assessed the age of onset (AOO), classified the ALXDRD type based on Russo’s (Infantile, Juvenile, Adult)^1^ and Prust’s (Type I, Type II)^3^ classifications, and documented the genetic testing methods, DNA source, clinical and radiological findings, inheritance patterns (*de novo* or inherited), as well as participant demographics including sex and country of origin. The genetic test applied and the source of DNA, initially recorded, were ultimately removed from the final table due to the presence of too many missing values. Finally, clinical and radiological symptoms were considered in the descriptive statistics only in relation to the Arg-substitutions (see below).

Among the 182 different variants identified, some were widely represented in the case series (i.e. p.Arg239Cys, p.Arg88Cys, p.Arg416Trp) while others were private variants found in only one participant (i.e. p.Arg283Ser, p.Arg284Tyr). Furthermore, among the 432 codons of the full-length *GFAP*, only 110 were affected by pathogenic variants and among these, 78 were unique variants (i.e. p.Arg283Ser) while the remaining 32 had different nucleotide changes within the same codon, thus producing alternative amino acid changes (i.e. p.Arg239Cys, p.Arg239His, p.Arg239Gly, p.Arg239Pro).

The distribution of causative variants in the *GFAP* gene among the 550 participants with ALXDRD is not uniform across its subdomains: 35.3% in coil 1A, 23.3% in coil 2B, 16.2% in coil 2A, 15.1% in the C-terminal tail domain, 6.4% in coil 1B and the remaining 3.7% in the linker regions L1-L12-L2 and N-terminal head domain. Regarding the coding position of these variants, the most frequently affected exons are: 40.9% exon 1, 20.7% exon 4, 14.9% exon 6 and 9.3% exon 8. The variants in the study are predominantly missense, accounting for 94.7% of the total. The most frequently altered amino acid is arginine (Arg), representing 49.6% of changes, followed by glutamic acid (10.7%), and leucine (7.8%). These variants are observed across all three ALXDRD AOO groups—infantile, juvenile, and adult—, while other variants, like p.Glu373Lys, are exclusive to a specific ALXDRD group.

### Participating patients retrieved

The participant demographics show a slight male predominance, with 52.2% males compared to 43.1% females (*p* < 0.01), while sex is unspecified for 4.7% of cases. Information on the country of origin of the patients is available for only 43.6% of cases, with 50.0% of these patients having European ancestry and 45.0% Asian. This distribution aligns with the nationality of the first authors in the 186 reviewed articles, where 46.8% are Asian, 31.0% European, and 20.0% North American. Notably, the combined percentage of European and North American authors is 51.0%, which is comparable to the 50% of patients with European ancestry.

Patients are classified by AOO into infantile (0–2 years), juvenile (3–12 years), and adult (> 12 years), and more recently by symptomatology into Type I and Type II. Although the symptom-based classification is gaining preference, it is absent in 87.6% of reports, whereas the AOO classification is missing in only 4.2%. The distribution among the reported classifications is 36.5% infantile, 18.4% juvenile, 40.9% adult, as well as 1.8% Type I, 10.4% Type II, and 0.2% Type III.

Regarding the genetic variants, 28.9% are identified as *de novo*, 11.1% are inherited, and the occurrence is unknown in 60% of cases. Of the inherited variants, mostly are maternally transmitted (60.7%), compared to 24.6% that are paternally transmitted (*p* < 0.01), while 1.6% are from both parents (homozygous variants), and in 13.1% of cases, the parental transmission is not documented.

### Amino acid frequency, age of onset and inheritance of ALXDRD

**Fig. 2:**
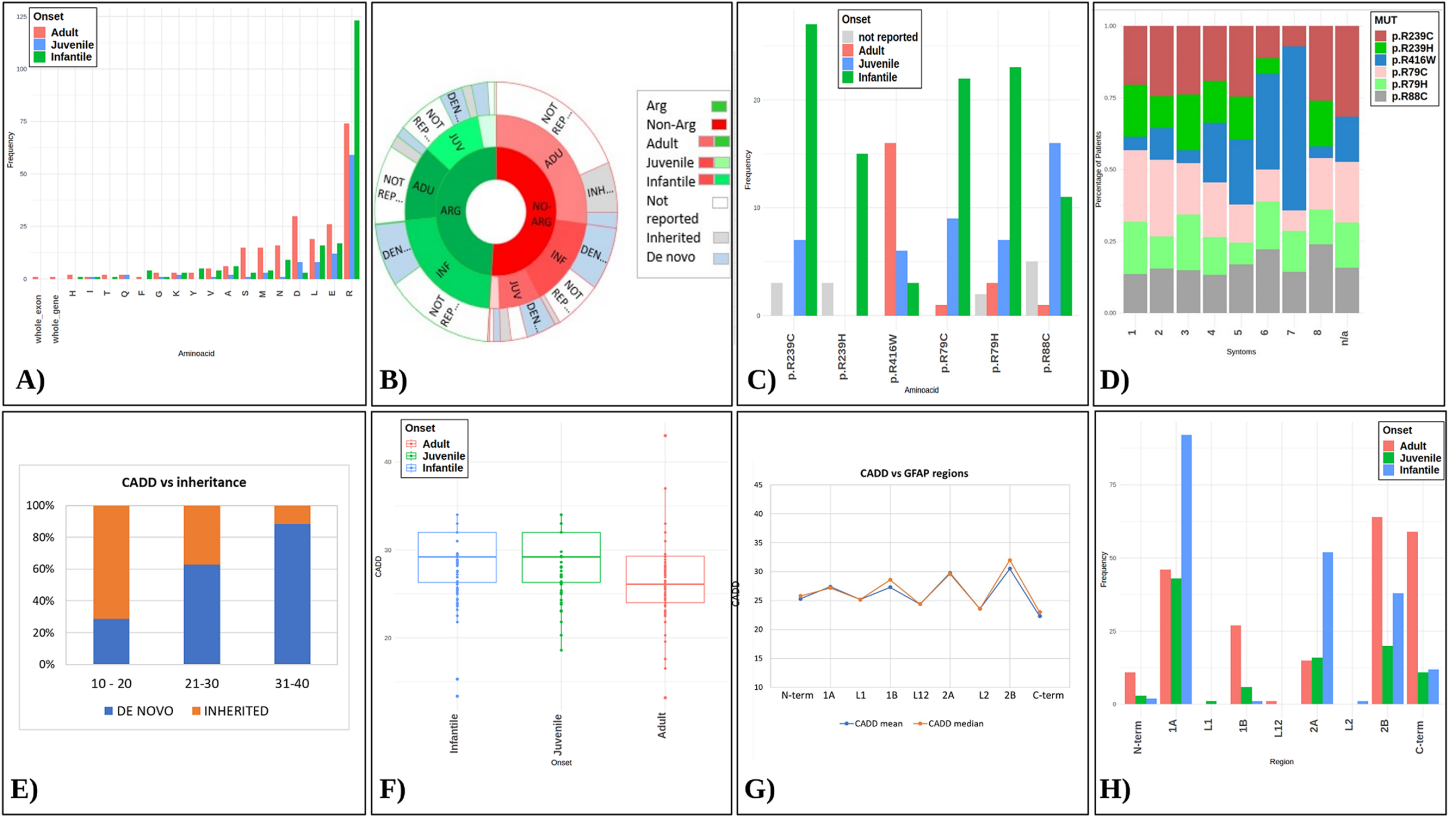
Relations between *GFAP* variants, Age of onset, CADD score and inheritance. (**A**) The barplot illustrates the amino acid predominantly affected in each of the three age of onset (AOO) categories, represented by light red for adult, light blue for juvenile, and light green for infantile. (**B**) The radial plot compares the mutated amino acids, reported as Arg *versus* non-Arg variants, in terms of associated AOO and occurrence. Almost half are arginine variants, prevalent in infantile cases, followed by adults and juveniles, with some missing data on age of onset. (**C**) Barplots of the six most prevalent variants at the Arg codon and their prevalence in different ALXDRD AOO. (**D**) Clinical data for 161 patients with the six prevalent Arg variants, categorized by EMBL-EBI Ontology (1. Neurodevelopmental abnormality; 2. Abnormality of higher mental function; 3. Seizure; 4. Abnormal central motor function; 5. Bulbar signs; 6. Other brainstem signs; 7. Somatic sensory dysfunction and abnormality of the autonomic nervous system; 8. Phenotypic abnormality). Notably, the distribution of symptoms varies among the different 6 most prevalent variants. (**E**) The rate of the *de novo* variants progressively increases with increasing CADD score, as shown in the bar graph for three different CADD ranges; conversely, inherited variants are progressively less represented with increasing CADD score range. (**F**) The distribution of CADD values with respect to age of onset shows that infantile and juvenile variants, represented in the box plots, have similar scores, on average higher than adults. (**G**) The mean (blue) and median (orange) CADD values were also stratified according to the position of the corresponding variants along the *GFAP* gene from N-terminal to C-terminal ends. Lines highlights the up-down alternating trend between secondary structured regions (1A-1B-2A-2B), belonging to the rod domain, and non-structured regions (C- and N-term and the L1, L12, L2 linkers). (**H**) Patients were also stratified by age of onset and position of the corresponding variant along the *GFAP* gene: infantile and adult cases present multiple variants in regions 1A and 2A, and in regions 2B and C-terminal, respectively.

Among the 550 ALXDRD patients, arginine is the most frequently mutated amino acid across all AOO categories, with 125 infantile, 59 juvenile, and 71 adults cases, and 18 in patients with unspecified age of onset (Fig. 2A). The 273 mutated Arg residues represent 49.6% of the total variants examined, making it the predominant variant. The other 277 variants, collectively referred to as non-Arg, impact a broad range of amino acids, except cysteine, proline, and tryptophan. Furthermore, two cases of copy number variations were noted: one involving exon 5 deletion^19^ and another a complete deletion of the *GFAP* gene^20^. When comparing the Arg and non-Arg variant categories, Arg variants are more prevalent in the infantile group (45.8%, *p* < 0.05) than in the juvenile (21.6%) and adult (26%) groups, with 6.6% having unspecified AOO. Conversely, non-Arg variants occur more frequently in adults (53.4%, *p* < 0.05) compared to juvenile (15.2%) and infantile (28.2%) groups, with 3.2% unspecified (Fig. 2B and Supplementary Table S3). Among the Arg variants, six are particularly prevalent: p.Arg239Cys (37 cases, 6.7% of total variants, 13.5% of Arg variants), p.Arg79His (35 cases, 6.4% of total, 12.8% of Arg), p.Arg88Cys (33 cases, 6% of total, 12% of Arg), p.Arg79Cys (32 cases, 5.8% of total, 11.7% of Arg), p.Arg416Trp (25 cases, 4.5% of total, 9.1% of Arg), and p.Arg239His (18 cases, 3.3% of total, 6.6% of Arg) (Fig. 2C). Notably, p.Arg239Cys is predominantly found in infantile cases, p.Arg88Cys in juvenile, and p.Arg416Trp in adult patients (*p* < 0.01) (Fig. 2C).

### Clinical focus on Arg variants

Clinical information is available for 178 of the 550 patients with ALXDRD reported in the present database (Supplementary Table S2). To attempt a first correlation between the genotypes at the *GFAP* locus and clinical signs, we focused on 161 out of the 180 patients identified with the six most common Arginine affecting variants: p.Arg239Cys, p.Arg79His, p.Arg88Cys, p.Arg79Cys, p.Arg416Trp, and p.Arg239His. Overall, these variants are associated with symptoms, classified using the EMBL-EBI Ontology Lookup Service (https://www.ebi.ac.uk/ols/index*)* (see Supplementary Clinical Information), across all defined categories, albeit with certain exceptions. For instance, symptoms under category 7, “somatic sensory dysfunction and anomalies of the autonomic nervous system”, have not been observed in patients with the p.Arg239His variant. Conversely, these symptoms are prevalent in individuals with the p.Arg416Trp variant, affecting 37.5% of these patients (Fig. 2D).

### CADD score, age of onset, and inheritance of ALXDRD

Combined Annotation Dependent Depletion (CADD) integrates multiple annotations into one metric for scoring the deleteriousness of single nucleotide variants and insertion/deletion variants in the human genome. A CADD score is a ranking, not a prediction, so low values indicate that the variant is biologically benign while high values, usually > 20, indicate that the variant is more likely to be biologically deleterious^21^. CADD scores were analyzed for the 540 SNVs identified in this systematic review, and assigned to three intervals: 0–20, 21–30, and 31–43. Given that some CADD values significantly deviated from the median value (median = 27.60, with min = 2.23 and max = 43.00), we applied the Rosner outlier test^22^, which resulted in the exclusion of the CADD value of 2.23 (*p* < 0.01).

By examining the inheritance information, available for only 219 of the total 540 SNV variants, and distributing the CADD scores of *de novo* and inherited variants into three intervals (10–20, 21–30, 31–40), it was observed that *de novo* variants typically exhibit higher mean/median CADD values than inherited variants (Supplementary Fig. S1). Furthermore, there is a trend such that as CADD scores increase, the rate of *de novo* variants increases while that of inherited variants decreases (Fig. 2E).

Specifically, in the 10–20 CADD range, there is a variant distribution of almost 30% *de novo* and 70% inherited, in contrast to the 31–40 CADD range, where 90% of the variants are *de novo*. A notable correlation was also identified between decreasing AOO and rising CADD scores, and vice versa (Supplementary Fig. S1). Moreover, the CADD score distribution relative to AOO indicates that infantile and juvenile variants typically have higher scores than adult variants (Fig. 2F), aligning with the higher proportion of inherited variants observed in adult cases (Supplementary Fig. S1).

Interestingly, the highest CADD values (see range 35–43 in Supplementary Fig. S2) correspond to variants identified exclusively in adult patients. Further examination of these variants revealed that, except for the nonsense pathogenic variant p.Glu312*, all are situated within ± 3 base pairs of exon-exon junctions, suggesting a potential impact on splicing.

### CADD score, age of onset, and GFAP domains

The CADD values were further stratified based on the location of the corresponding variants within the *GFAP* gene (Fig. 2G), observing that the mean and median CADD scores are generally lower in the flanking N-terminal head and C-terminal tail regions and in the three linker regions of the rod domain than the four rod coils 1A, 1B, 2A and 2B, with the highest values noted in the 2B coil (Fig. 2G).

A potential correlation between the location of the variants within the *GFAP* gene and the AOO of the disease was revealed by distinct associations: infantile and adult cases predominantly exhibit variants in the 1A and 2A coils, and in the 2B coil and tail domain, respectively (Fig. 2H).

### CADD score, age of onset and amino acid changes

**Fig. 3:**
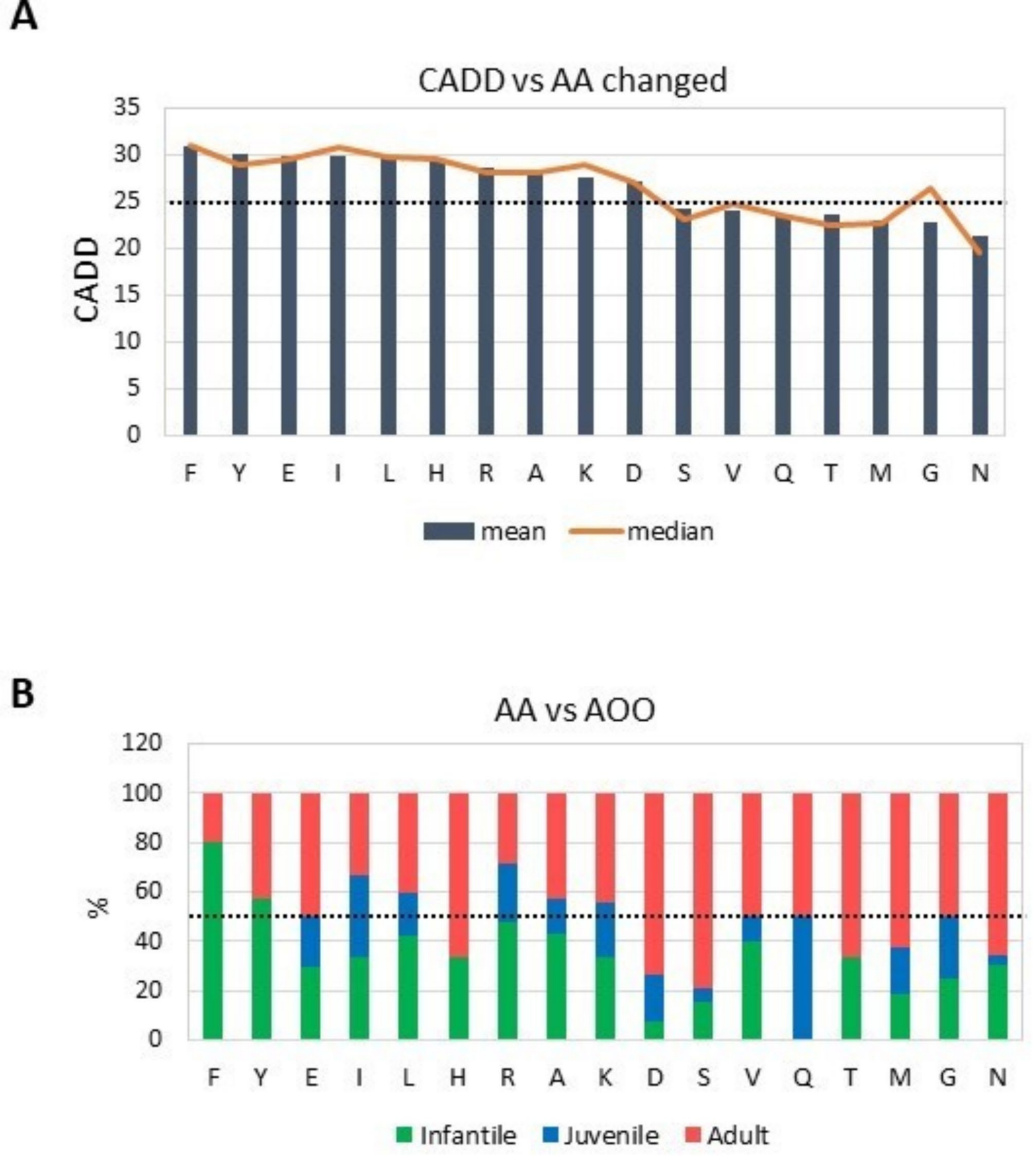
Focus on AA changes and their relations with CADD and Age of onset. (**A**) The graph represents the mean (dark blue bars) and median (orange line) CADD values for each amino acid (AA) that is affected in the surveyed variants of the *GFAP* gene. Values are represented from highest to lowest. The dotted line indicates the CADD 25 cut-off. (**B**). In the graph, each bar corresponds to an AA affected by a *GFAP* variant. For each bar, the percentage of infantile (light green), juvenile (light blue), and adult (light red) patients carrying a variant that modifies this AA is represented. The dotted line indicates the percentage of 50% of total cases.

Variants with a CADD score greater than 25 predominantly induce amino acid replacements in the following descending order of score: glutamic acid, phenylalanine, histidine, isoleucine, leucine, arginine, lysine, aspartic acid, and tyrosine (Fig. 3A). Furthermore, the analysis of amino acid changes in relation to AOO reveals that phenylalanine and tyrosine substitutions are major contributors to infantile ALXDRD, accounting for over 50% of cases. Conversely, glutamine substitutions are evenly distributed between juvenile and adult cases, while serine and aspartic acid changes are predominantly associated with adult ALXDRD (Fig. 3B).

A significant observation is that 7 out of 9 variants (77.8%) that alter amino acids with a mean CADD score below 25 (Ser, Val, Gln, Thr, Met, Asn, Gly) correlate with adult AOO in at least 50% of cases (Fig. 3B).

In line with recent findings, which linked specific motifs in the rod domains to GFAP assembly defects^23^, our review identified 47 out of 550 cases (8.5%) involving leucine (Leu) substitutions, with 30 of these being replaced by proline (Pro). Notably, 10 of these substitutions were observed in infantile patients, including 5 at birth (0.01 years in Supplementary Table S2). Among the 17 variants linked to disease onset at birth, 7 impacted arginine (Arg) residues, underscoring that changes in leucine (Leu) and arginine (Arg) are associated with the most severe phenotypes (data not shown).

### ACMG classification, CADD score and age of onset of ALXDRD

According to the American College fo Medical Genetics (ACMG) criteria, variants are classified into five tiers: Pathogenic (P), Likely pathogenic (LP), Uncertain significance (VUS), Likely benign (LB), and Benign (B), depending on the weight and type of evidence: population, computational, functional, segregation, *de novo*, allelic, other database, and other data^24^. The ClinVar database classifies variants according their potential clinical impact using the ACMG criteria. To evaluate further the size effect of variants, ClinVar classifìcation was retrieved for each patient variant from the Clinvar annotated VCF file, downloaded from https://ftp.ncbi.nlm.nih.gov/pub/clinvar/vcf_GRCh38/ on Sept 11th, 2024. The effect of 232 out of 550 patient variants could be predicted, while the remaining 318 variants were (i) “not provided”, (ii) characterized by “conflicting classification of pathogenicity”, or (iii) “NA”, namely variants unreported in the database. Among these 232 variants, classified into one of three categories (B + LB; VUS; LP + P), only 223 could be correlated with the age of disease onset which in fact was not available for 9 variants (Supplementary Fig. S3). As expected, excluding the few B/LB variants whose presence is very limited (9 out of 223) and their distribution quite random, the VUS variants show a decrease going from adult, juvenile to infantile cases (r^2^ = 0.9915, *p* = 0.0584), with the P/LP variants having, as expected, an opposite trend (r^2^ = 0.9350, *p* = 0.1641). The CADD values for the three classes were also averaged and found to correlate very well with increased clinical pathogenicity (r^2^ = 0.3294, *p* = 0.0114) (Supplementary Fig. S3).

### Meta-analysis

Based on the findings of our systematic review, we conducted a meta-analysis focusing on specific subgroups to assess the prevalence of certain variants across different patients. Specifically, prompted by the higher incidence of Arg variants in infantile onset cases, we evaluated the prevalence of the p.Arg416Trp and p.Arg88Cys/Ser variants in infants compared to a combined group of juveniles and adults (Fig. 2A). Then, we compared Arg variants against non-Arg variants, in both infant and adult cases. In the figure we report the results from the REML model, particularly useful to manage the eterogeneity beetwen studies with small sample size.

#### p.Arg416Trp in infantile versus juvenile/adult subgroup

**Fig. 4:**
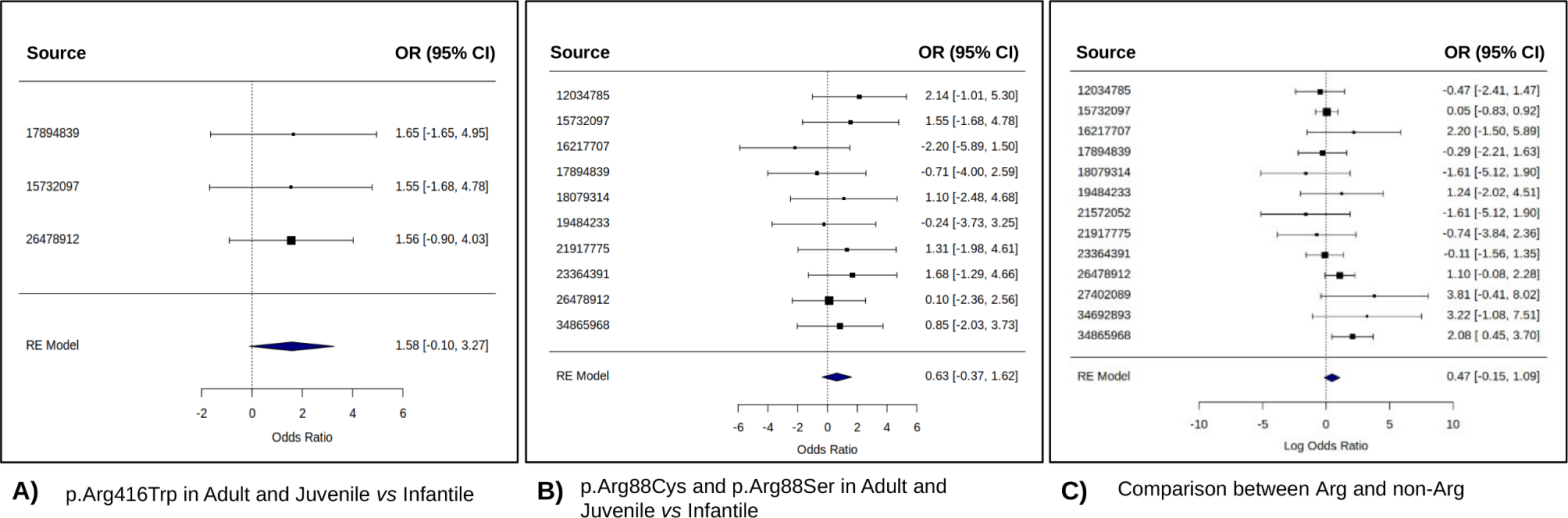
Forest Plots for *GFAP* variants vs. age of onset associations using the REML model. (**A**) The meta-analysis compares studies reporting the p.Arg416Trp variant between adult, juvenile, and infantile patients. (**B**) Results from the meta-analysis comparing studies reporting the p.Arg88Cys and p.Arg88Ser variants between adult, juvenile, and infantile patients. (**C**) Comparison of the effects induced by Arg (arginine) and non-Arg variants focused mainly on disease progression.

Three studies were considered to evaluate the p.Arg416Trp variant. Using metabin package with the Random-Effects Model, we observed minimal total heterogeneity (I^2^ = 0.00%, H^2^ = 1.00), with the heterogeneity test indicative of non-significant variation (*p* = 0.9989), i.e. consistent results across studies.The analysis yielded an odds ratio (OR) of 1.5822 with a 95% confidence interval (CI) ranging from − 0.1018 to 3.2662. Despite a p-value of 0.0655, which falls short of conventional significance, there appears to be a trend towards a potential association (Fig. 4A).

#### p.Arg88Cys/Ser in infantile versus juvenile/adult subgroups

Considering 10 studies with 328 observations and 20 events, we used metabin package to compare the study subgroups: the Common Effect Model estimated an OR of 2.0086 (95%-CI: [0.8288; 4.8677]) for the p.Arg88Cys/Ser, while the Random Effects Model provided an OR of 2.0502 (95%-CI: [0.7470; 5.6268]). Both models showed no significant association, as indicated by non-significant p-values of 0.1225 and 0.1634, respectively. The heterogeneity tests for both common and random effects model confirmed low variability (I^2^ = 0.0%, H^2^ = 1.00), suggesting a consistency pattern across the included studies (Fig. 4B).

#### Arginine versus non-arginine variants

This meta-analysis included 13 studies to explore differences in prevalence between Arg and non-Arg variants. The random effects model with rme package estimated a tau² of 0.2304 (SE = 0.4401), with I² at 19.37% and H² at 1.24, indicating moderate heterogeneity. The p-value for heterogeneity was 0.1835. With more than ten studies included, a funnel plot was constructed to detect publication bias, which appeared symmetric (Supplementary Fig. S4), implying a balanced dataset and reducing concerns about publication bias (Fig. 4C).

## Discussion

Alexander disease is a rare neurodegenerative disorder resulting from pathogenic variants in the *GFAP* gene. Establishing genotype-phenotype correlations remains challenging due to reduced penetrance and variable expressivity of clinical manifestations, observed even among individuals carrying the same genetic variant.

By integrating data from various papers, meta-analyses can enhance statistical power, validate associations, and provide evidence to elucidate the genetic basis of diseases. After assessing heterogeneity across multiple studies, meta-analyses allowed us to determine the effects of *GFAP* gene variants, thus strengthening the significance of the reported associations, and confirming the usefulness of this approach when individual studies may not provide sufficient cases and/or controls to draw definitive conclusions.

First of all, an unbiased collection of *GFAP* variants, identified in patients with ALXDRD from January 2001, when the *GFAP* gene was first implicated in the condition^25^, to December 29, 2023, was realized. Specifically, our focus has been on the structure of the *GFAP* gene and protein, genetic information such as the inheritance pattern (inherited or *de novo*) and CADD scores for deleteriousness assessment, variant types, and the AOO in ALXDRD patients, alongside their clinical manifestations. Although a recent classification of ALXDRD into Types I and II has been proposed^3,4^, we have opted to utilize the earlier classification system based on AOO^1^. This approach not only reflects the disease severity, but is also the methodology adopted by most of the studies we reviewed. Moreover, as a measure of pathogenicity we collected data on the ACMG classification of each of the *GFAP* variant under consideration. Although the ClinVar classification could only be retrieved for 223 variants, these were sufficient to draw conclusions about correlation with age at onset and corresponding CADD values. These results confirmed what we expected and in particular that both CADD and ClinVar (i.e. the ACMG criteria) are useful means to predict the clinical impact and deleteriousness of *GFAP* variants in ALXDRD.

We identified 550 causative *GFAP* variants, predominantly missense. Arginine is the residue most frequently altered, followed by glutamic acid and leucine. The high pathogenic variants rate of these residues aligns with their common presence in alpha-helix structures, which are crucial to the architecture of intermediate filaments^26^. However, the significance of Arginine extends beyond its role in alpha-helices; in an analysis of 44 proteins associated with genetic diseases, arginine was found to be the most frequently mutated residue across various secondary structures^27^.

Arginine variants are more prevalent in early-onset forms of ALXDRD, underscoring the essential role of this amino acid in GFAP protein assembly and function. Typically, Arg variants arise *de novo*, particularly in severe early-onset cases. Conversely, non-Arg changes are more common in adult-onset ALXDRD, and are often inherited as an autosomal dominant trait. In fact, in these cases, the risk of recurrence exists since the manifestation and progression of symptoms frequently occur beyond the reproductive years. Notably, the p.Arg416Trp variant, predominantly found in adult-onset ALXDRD forms, may relate to its unique impact on cellular aggregate formation compared to the p.Arg239Cys variant, which is primarily observed in infantile cases, probably due to the different domains affected, as highlighted through the real-time evaluation of aggregate formation dynamics^28^. The papers reporting data on Arg variants were also used to run meta-analyses that provided a quantitative assessment of the comparisons confirming consistent effect sizes with minimal heterogeneity.

From a structural perspective, our analysis revealed that CADD values are notably higher for variants altering residues within the coils of the rod domain (i.e., the 1A, 1B, 2A, and 2B coils) compared to those occurring in the non-alpha-helical N-terminal (head), C-terminal (tail) and linker regions of the rod domain (L1, L12, and L2). This suggests that amino acid changes in the rod domain have a more significant impact on protein conformation. In contrast, variations in the N- and C-terminal regions, which exhibit lower CADD values, a higher percentage of inherited variants, and a predominance of adult-onset ALXDRD, indicate the greater structural tolerance of these regions, and reduced constraints to amino acid substitutions. This distinction underscores the differential impact of variants based on their location within the GFAP protein.

It has been shown that very high CADD values are linked to a subset of adult-onset ALXDRD patients, a phenomenon that could be attributed to potential altered splicing effects due to the proximity of the variants to exon-intron boundaries. This aligns with findings by Lord et al. (2019)^29^, who reported that 27% of splicing variants occur in non-canonical splicing sites, with changes from purine to pyrimidine at the last nucleotide of an exon being particularly deleterious. Indeed, for two specific variants, c.1127G > A (p.Arg376Leu) and c.1127G > T (p.Arg376Gln), located at the last nucleotide of *GFAP* exon 6, we identified aberrant splicing products. These products, leading to premature stop codons and potential nonsense-mediated decay (NMD), might explain the milder phenotypes seen with these high-impact missense variants (Bachetti et al., in preparation). Such a mechanism could act protectively, limiting the penetrance of severe variants, similar to the down-regulation observed with RNA interference technologies like antisense methods^30^ which specifically target the mutant allele. In fact, in the case of ALXDRD, the reduced mutant protein production, compared to normal mRNA splicing, could delay disease onset. This is also why the highest CADD score (43) was assigned to the p.Glu312* nonsense variant, which introduces a stop codon in exon 6 likely triggering NMD, associated with an adult ALXDRD case. In contrast, the frameshift variant p.Val431Aspfs*14, found in an infantile ALXDRD case and located at the gene second last codon, may evade NMD, allowing full production of an aberrantly elongated mutant protein. Moreover, among 19 frameshift variants, 10 (52,6%) were identified in adult-onset patients, while 4 (21%) and 5 (26,3%) were found in juvenile and infantile groups, respectively. Notably, frameshifts associated with infantile ALXDRD predominantly occur in the last two exons of the *GFAP* gene, where NMD escape is more probable. In contrast, 50% of frameshift variants found in adult-onset patients are located within the 5 bp interval around the canonical GT(5’)-AG(3’) intronic splice site, suggesting a relationship between variant location, splicing, and disease onset.

As a result of these observations, CADD does not appear to be reliable in predicting the clinical effect of the variant in causing ALXDRD disease, i.e., a GFAP null allele may not be pathogenic even if it is predicted to be deleterious by the CADD score. However, we suggest that such apparent inconsistencies may reveal new molecular mechanisms underlying unexpected associations between CADD values and clinical phenotypes.In addition to post-transcriptional mechanisms such as the splicing process, the impact of *GFAP* variants on the AOO of ALXDRD may be influenced by the structural roles of the amino acids that are substituted. Indeed, we observed that replacing different amino acids at the same codon in the GFAP protein can result in different AOOs. For example, the p.Arg239Cys variant has not been found in adult patients, while the p.Arg239Gly variant does not occur in infantile cases, suggesting that the specific amino acid change at position 239 can influence the timing of symptoms onset. Interestingly, even though glycine substitutions can disrupt alpha-helix stability—a crucial element for protein function^31^—the only known infantile case involving arginine to glycine (R to G) changes is associated with the p.Arg79Gly variant while Arg to Gly substitutions are mostly seen in adults ALXDRD. This observation underlines that both the nature of the amino acid substitution and its specific location within the protein may be determinants of when the symptoms of Alexander disease will manifest.

Moreover, although the limited number of specific variants significantly constrains our ability to establish statistically significant associations, p.Glu373Lys and p.Ser385Phe are exclusively found in infantile ALXDRD cases, whereas p.Glu373Ala and p.Ser385Cys are predominantly observed in adults. Additionally, certain *GFAP* variants have been uniquely identified in infantile or adult patients, suggesting the possibility that some *GFAP-*specific changes may trigger distinct pathogenetic mechanisms. Furthermore, in the light of the elusive genotype-phenotype correlation for distinguishing different forms of the disease, the effect of genetic modifiers, either located within the *GFAP* gene itself or in other interacting genes, possibly accounting for variable expressivity of ALXDRD cannot be ruled out at this stage.

Unlike the initially reported high prevalence of infantile cases^13^, our observations reveal a substantial proportion of adult ALXDRD patients, which allowed us to identify previously overlooked correlations. This shift could be attributed to heightened awareness and recognition of ALXDRD in the differential diagnosis of other more prevalent neurodegenerative disorders.

Finally, a gender issue emerged, both in the male-female relationship and between fathers and mothers who transmitted the causative variant. In fact, it seems that while males are more affected, mothers more frequently transmit the variant they carry (inherited variants) and therefore the disease. In the past, Li et al. (2006)^32^ had already found that the *GFAP* causative variants occurred in one of the parental germ lines (*de novo* variants) had originated predominantly on the paternal chromosome (*P* < 0.001), during spermatogenesis. Unfortunately, the explanation for why the inherited *GFAP* variants are predominantly of maternal origin is not known, even if this is a phenomenon already observed for the transmission of other gene variants in different diseases^33^.

In conclusion, our systematic review, followed by meta-analysis, has revealed previously unrecognized correlations in ALXDRD. The CADD score appears to be a reliable indicator of the impact of *GFAP* pathogenic variants and, when discrepancies between the CADD score and the age of onset (AOO) or clinical severity are observed, further research are suggested to elucidate underlying pathogenetic mechanisms, such as aberrant splicing, which could play an unexpected role in the manifestation of the disease.

## Methods

This review was undertaken and is reported in accordance with the Preferred Reporting Items for Systematic review and Meta-Analyses (PRISMA) guidelines (https://www.bmj.com/content/372/bmj.n71).

### Systematic literature search and eligibility criteria

We systematically searched relevant literature databases, including PubMed, Web of Science and Waisman Center, using the following key-words: (glial fibrillary acidic protein AND Alexander’s disease) OR (glial fibrillary acidic protein AND Alexander disease) OR (GFAP AND Alexander Disease) OR (GFAP AND Alexander’s Disease).

Published case reports and articles of proven genetic diagnosis of Alexander Disease, published in peer-reviewed journals in English, were reviewed. To be included, articles had to report at least one novel case, and to provide the following information: (i) details on the genetic variant(s) and clinical symptoms associated with reported ALXDRD patient(s); (ii) type of ALXDRD (infantile or juvenile/adult) or age of onset.

We excluded conference abstracts, editorials, papers on unconfirmed genetic diagnosis of ALXDRD, papers on animal models, review articles without description of new patients, or case series without genetic data or with genetics reported incorrectly.

The searches were limited to human studies published from January 2001 to December 29, 2023. Further relevant articles were also searched in the bibliography lists of the papers already selected, thus allowing additional articles to be included.

### Data screening and extraction

All records were screened for eligibility at the title/abstract level and then at the full-text level.

After removal of duplicates, the remaining titles and abstracts were assessed for inclusion. The full texts of relevant articles were retrieved and independently assessed by two authors (A.G. and F.R.). Standardized extraction forms were used for the data collection process. Data were extracted independently by the two authors above and compared. The discrepancies were discussed with I.C. and T.B. as judges. Data extracted included year of publication, participant demographics, diagnosis (including method of diagnosis and source of DNA). Articles without single-nucleotide variations or insertion/deletions of the *GFAP* gene were excluded. Nomenclature for all variants was confirmed and reported using canonical transcripts in VarSome20, Ensembl Genome Browser, and dbSNP.

Unpublished data from the laboratory of Genetics and Genomics of Rare Diseases of the IRCCS Giannina Gaslini Institute were also included.

### Summary measures and statistical analysis

Data analysis was conducted using Jamovi 2.3 (retrieved from https://www.jamovi.org*)* and the R 4.2.2 software^34^. Patient features, disease manifestations, and variant characterization were reported descriptively. Categorical variables were evaluated by chi-squared test or Fisher’s exact test. Spearman correlation between CADD (Combined Annotation Dependent Depletion, GRCh38-v1.6) score (https://cadd.gs.washington.edu/snv*)* and ALXDRD onset was used to determine the pathogenic risk for the single nucleotide variants (SNVs). A p-value < 0.05 was statistically significant.

### Meta-analyses

The meta-analyses were performed with R, using metafor and metabin packages^35^. We applied the random-effect model, with the DerSimonian and Laird method^36^. We chose to use the Mantel-Haenszel method^37^ for pooling odds ratios (ORs) under a fixed-effect model, and also compare the results with a random-effects model using the Restricted Maximum Likelihood (REML) estimator to account for potential heterogeneity across studies. For all analyses, between-study heterogeneity was assessed using the between-study variance (τ2), the I^2^ statistic and Cochran’s Q test I^2^. Subgroup analyses were conducted to minimize severe heterogeneity between studies, where an I² value below 50% indicated low heterogeneity. Subgroup differences in rates, ORs and 95% confidence intervals (CIs) were tested for each comparison. Forest plots were used to summarize statistics from individual study and pooled group meta-analysis. Funnel plots were tested to estimate publication bias.

### Outcomes

The main findings resulted from the genetic and clinical data reported in each study. In particular, the genetic alterations present in proband(s) and siblings, offspring, and parents of the proband(s) were used, including type of alterations (e.g. insertion, deletion, SNV) and pathogenic effects. As for clinical data, age of onset and symptoms were taken into consideration.

### Risk of bias

Performance biases and selective reporting could potentially influence the statistical analysis of the gene variants and associated clinics. The OHAT Risk of Bias Rating Tool for Human and Animal Studies (https://hsls.libguides.com/reporting-study-tools/risk-of-bias*)* allowed a self-assessment by asking “Were experimental conditions identical across study groups?” and “Were all measured outcomes reported?”. Regarding the first question, the answers “Definitely Low” and “Probably Low” were found adequate among those suggested by the tool since there is indirect evidence that the same conditions were used in control and case samples for the sequencing analysis. As for the second question, we provided two answers: “Probably Low”, based on indirect evidence that all measured outcomes of the study, such as genetic diagnosis, were reported, and “Probably High”, based on the fact that other related, secondary outcomes such as onset, age or the presence of specific symptoms are not always reported in all the papers considered.

## Electronic supplementary material

Below is the link to the electronic supplementary material.

Supplementary Material 1 (Suppl. Clinical Information)

Supplementary Material 2 (Tables S1, S2, and S3)

Supplementary Material 3 (Figures S1-S4)

## Data Availability

All data generated or analysed during this study are included in this published article (and its Supplementary Information files).

## Abbreviations

ALXDRD: Alexander Disease
GFAP: Glial Fibrillary Acidic Protein
AOO: Age of onset
sHSPs: Small heat shock proteins
SNV: Single nucleotide variant
CADD: Combined Annotation Dependent Depletion

## References

[1] Russo, L. S., Aron, A. & Anderson, P. J. Alexander’s disease: a report and reappraisal. *Neurol*. **26**, 607–614 (1976). 10.1212/wnl.26.7.607 180453

[2] Springer, S. et al. Alexander disease–classification revisited and isolation of a neonatal form. *Neuropediatrics*. **31**, 86–92 (2000). 10832583 10.1055/s-2000-7479

[3] Prust, M. et al. GFAP mutations, age at onset, and clinical subtypes in Alexander disease. *Neurol*. **77**, 1287–1294 (2011). 10.1212/WNL.0b013e3182309f72 PMC3179649 21917775

[4] Yoshida, T. et al. Nationwide survey of Alexander disease in Japan and proposed new guidelines for diagnosis. *J. Neurol. ***258**, 1998–2008 (2011). 21533827 10.1007/s00415-011-6056-3

[5] Nielsen, A. L., Jørgensen, P. & Jørgensen, A. L. Mutations associated with a childhood leukodystrophy, Alexander disease, cause deficiency in dimerization of the cytoskeletal protein GFAP. *J. Neurogenet. ***16**, 175–179 (2002). 12696672 10.1080/01677060215305

[6] Tang, G., Perng, M. D., Wilk, S., Quinlan, R. & Goldman, J. E. Oligomers of mutant glial fibrillary acidic protein (GFAP) inhibit the proteasome system in alexander disease astrocytes, and the small heat shock protein alphab-crystallin reverses the inhibition. *J. Biol. Chem. ***285**, 10527–10537 (2010). 20110364 10.1074/jbc.M109.067975 PMC2856260

[7] Hagemann, T. L., Boelens, W. C., Wawrousek, E. F. & Messing, A. Suppression of GFAP toxicity by alphab-crystallin in mouse models of Alexander disease. *Hum. Mol. Genet. ***18**, 1190–1199 (2009). 19129171 10.1093/hmg/ddp013 PMC2655774

[8] Shigetomi, E., Saito, K., Sano, F. & Koizumi, S. Aberrant calcium signals in reactive astrocytes: a key process in neurological disorders. *Int. J. Mol. Sci. ***20**, 996 (2019). 30823575 10.3390/ijms20040996 PMC6413203

[9] Sosunov, A. A., McKhann, G. M. & Goldman, J. E. The origin of Rosenthal fibers and their contributions to astrocyte pathology in Alexander disease. *Acta Neuropathol. Commun. ***5**, 27 (2017). 28359321 10.1186/s40478-017-0425-9 PMC5374671

[10] Heaven, M. R. et al. Composition of Rosenthal Fibers, the protein Aggregate Hallmark of Alexander Disease. *J. Proteome Res. ***15**, 2265–2282 (2016). 27193225 10.1021/acs.jproteome.6b00316 PMC5036859

[11] Bachetti, T. et al. A novel polymorphic AP-1 binding element of the GFAP promoter is associated with different allelic transcriptional activities. *Ann. Hum. Genet. ***74**, 506–515 (2010). 20946255 10.1111/j.1469-1809.2010.00614.x

[12] Hagemann, T. L. et al. Antisense therapy in a rat model of Alexander disease reverses GFAP pathology, white matter deficits, and motor impairment. *Sci. Transl Med. ***13**, 620 (2021). 10.1126/scitranslmed.abg4711 PMC8730534 34788075

[13] Messing, A. & Brenner, M. G. F. A. P. Functional implications gleaned from studies of genetically engineered mice. *Glia*. **43**, 87–90 (2003). 12761871 10.1002/glia.10219

[14] Quinlan, R. A., Brenner, M., Goldman, J. E. & Messing, A. GFAP and its role in Alexander disease. *Exp. Cell. Res. ***313**, 2077–2087 (2007). 17498694 10.1016/j.yexcr.2007.04.004 PMC2702672

[15] Balbi, P. et al. The clinical spectrum of late-onset Alexander disease: a systematic literature review. *J. Neurol. ***257**, 1955–1962 (2010). 20721574 10.1007/s00415-010-5706-1

[16] Heshmatzad, K. et al. GFAP variants leading to infantile Alexander disease: phenotype and genotype analysis of 135 cases and report of a de novo variant. *Clin. Neurol. Neurosurg. ***207**, 106754 (2021). 34146839 10.1016/j.clineuro.2021.106754

[17] Vaia, Y., Mura, E. & Tonduti, D. Type I Alexander disease: update and validation of the clinical evolution-based classification. *Mol. Genet. Metab. ***138**, 107540 (2023). 36804850 10.1016/j.ymgme.2023.107540

[18] Heshmatzad, K., Naderi, N., Masoumi, T., Pouraliakbar, H. & Kalayinia, S. Identification of a novel de novo pathogenic variant in GFAP in an Iranian family with Alexander disease by whole-exome sequencing. *Eur. J. Med. Res. ***27**, 174 (2022). 36088400 10.1186/s40001-022-00799-5 PMC9464415

[19] Graff-Radford, J. et al. The neuroanatomy of pure apraxia of speech in stroke. *Brain Lang. ***129**, 43–46 (2014). 24556336 10.1016/j.bandl.2014.01.004 PMC4004427

[20] Kim, M. H., Lee, J. S., Hong, J. M., Sohn, Y. B. & Lee, S. J. Aperiodic alternating nystagmus in adult-onset Alexander disease with a novel mutation. *J. Neurol. ***270**, 569–572 (2023). 36153801 10.1007/s00415-022-11390-7

[21] Kircher, M. et al. A general framework for estimating the relative pathogenicity of human genetic variants. *Nat. Genet. ***46** (3), 310–315 (2014). 24487276 10.1038/ng.2892 PMC3992975

[22] Rosner, B. Percentage points for a generalized ESD many-outlier Procedure. *Technometrics*. **25**, 165–172 (1983).

[23] Yang, A. W., Lin, N. H., Yeh, T. H., Snider, N. & Perng, M. D. Effects of Alexander disease–associated mutations on the assembly and organization of GFAP intermediate filaments. *Mol. Biol. Cell. ***33**, ar69 (2022). 35511821 10.1091/mbc.E22-01-0013 PMC9635275

[24] Richards, S. et al. Standards and guidelines for the interpretation of sequence variants: a joint consensus recommendation of the American College of Medical Genetics and Genomics and the Association for Molecular Pathology. *Genet. Med. ***17** (5), 405–424 (2015). 25741868 10.1038/gim.2015.30 PMC4544753

[25] Brenner, M. et al. Mutations in GFAP, encoding glial fibrillary acidic protein, are associated with Alexander disease. *Nat. Genet. ***27**, 117–120 (2001). 11138011 10.1038/83679

[26] Prothero, J. W. A model of alpha-helical distribution in proteins. *Biophys. J. ***8**, 1236–1255 (1968). 5696210 10.1016/S0006-3495(68)86553-1 PMC1367692

[27] Khan, S. & Vihinen, M. Spectrum of disease-causing mutations in protein secondary structures. *BMC Struct. Biol. ***7**, 56 (2007). 17727703 10.1186/1472-6807-7-56 PMC1995201

[28] Kaneko, H. et al. Novel GFAP mutation in patient with adult-onset Alexander disease presenting with spastic ataxia. *Mov. Disord Off J. Mov. Disord Soc. ***24**, 1393–1395 (2009). 10.1002/mds.22556 19412928

[29] Lord, J. et al. Pathogenicity and selective constraint on variation near splice sites. *Genome Res. ***29**, 159–170 (2019). 30587507 10.1101/gr.238444.118 PMC6360807

[30] Hagemann, T. L. et al. Antisense suppression of glial fibrillary acidic protein as a treatment for Alexander disease. *Ann. Neurol. ***83**, 27–39 (2018). 29226998 10.1002/ana.25118 PMC5876100

[31] Aurora, R., Srinivasan, R. & Rose, G. D. Rules for alpha-helix termination by glycine. *Science*. **264**, 1126–1130 (1994). 8178170 10.1126/science.8178170

[32] Li, R. et al. Propensity for paternal inheritance of de novo mutations in Alexander disease. *Hum. Genet. ***119**, 137–144 (2006). 16365765 10.1007/s00439-005-0116-7

[33] Jannot, A. S. et al. Male and female differential reproductive rate could explain parental transmission asymmetry of mutation origin in Hirschsprung disease. *Eur. J. Hum. Genet. EJHG*. **20**, 917–920 (2012). 22395866 10.1038/ejhg.2012.35 PMC3421120

[34] R Core Team. R: A language and environment for statistical computing. V.4.2.2 (R Version 4.2.2). (2021). https://www.R-project.org/

[35] metabin function - RDocumentation. V.4.2.2 (R Version 4.2.2) https://www.rdocumentation.org/packages/meta/versions/6.5-0/topics/metabin

[36] DerSimonian, R. & Laird, N. Meta-analysis in clinical trials. *Control Clin. Trials*. **7**, 177–188 (1986). 3802833 10.1016/0197-2456(86)90046-2

[37] Mantel, N. & Haenszel, W. Statistical aspects of the analysis of data from retrospective studies of disease. *J. Natl. Cancer Inst. ***22**, 719–748 (1959). 13655060

