# Supplementary material for: A systematic review and meta-analysis of GFAP gene variants in Alexander disease
Source: Sci Rep. 2024 Oct 17;14:24341. doi: 10.1038/s41598-024-75383-4 (PMC11487261; doi:10.1038/s41598-024-75383-4)
Supplement: Supplementary file 1 — Supplementary Material 1 (Suppl. Clinical Information) [file 41598_2024_75383_MOESM1_ESM.pdf]

CLINICAL AND MRI FEATURES OF ALEXANDER DISEASE - https://www.ebi.ac.uk/ols/index

1. **NEURODEVELOPMENTAL ABNORMALITY** (http://purl.obolibrary.org/obo/HP_0012759)
   - Motor delay (http://purl.obolibrary.org/obo/HP_0001270)
   - Motor deterioration (http://purl.obolibrary.org/obo/HP_0002333)
   - Delayed speech and language development (http://purl.obolibrary.org/obo/HP_0000750)
   - Intellectual disability (http://purl.obolibrary.org/obo/HP_0001249)
   - Psychomotor retardation (http://purl.obolibrary.org/obo/HP_0025356)
   - Global developmental delay (http://purl.obolibrary.org/obo/HP_0001263)
   - Developmental stagnation (http://purl.obolibrary.org/obo/HP_0007281)
   - Poor trunk control (http://snomed.info/id/249855007)
   - Developmental regression (http://purl.obolibrary.org/obo/HP_0002376)

2. **ABNORMALITY OF HIGHER MENTAL FUNCTION (cognitive and behavioral abnormalities)**
   - Cognitive impairment (http://purl.obolibrary.org/obo/HP_0100543)
   - Abnormality of higher mental function (http://purl.obolibrary.org/obo/HP_0011446)
   - Absent speech (http://purl.obolibrary.org/obo/HP_0001344)
   - Encephalopathy (http://purl.obolibrary.org/obo/HP_0001298)
   - Hallucinations (http://purl.obolibrary.org/obo/HP_0000738)
   - Anorexia (http://purl.obolibrary.org/obo/HP_0002039)

3. **SEIZURE (& Hyperkinesia)**
   - Seizure (http://purl.obolibrary.org/obo/HP_0001250)
   - Arching back and eye rolling upward | Back arching (http://purl.obolibrary.org/obo/NCIT_C121556) (and eye rolling upwards)
   - Hyperkinesia (http://purl.obolibrary.org/obo/NCIT_C116575)
   - (Dyskinesia) (http://purl.obolibrary.org/obo/HP_0100660)

4. **ABNORMAL CENTRAL MOTOR FUNCTION** ((http://purl.obolibrary.org/obo/HP_0011442)
   - Gait disturbance (http://purl.obolibrary.org/obo/HP_0001288)
   - Inability to walk (http://purl.obolibrary.org/obo/HP_0002540)
   - Spasticity (http://purl.obolibrary.org/obo/HP_0001257)
   - Ataxia (http://purl.obolibrary.org/obo/HP_0001251)
   - Incoordination (http://purl.obolibrary.org/obo/HP_0002311)
   - Inability to walk (http://purl.obolibrary.org/obo/HP_0002540)
   - Balance coordination, abnormal motor coordination/balance (http://purl.obolibrary.org/obo/MP_0001516)
   - Weakness (Weakness due to upper motor neuron dysfunction - http://purl.obolibrary.org/obo/HP_0010549)
   - Poor head control (http://purl.obolibrary.org/obo/HP_0002421)
   - Hypotonia (central hypotonia | http://purl.obolibrary.org/obo/HP_0001252)
   - Hyperreflexia (http://purl.obolibrary.org/obo/HP_0001347)
   - Weak grip (http://purl.obolibrary.org/obo/HP_0033466)
   - Tremor (http://purl.obolibrary.org/obo/HP_0001337)
   - Fatigue (tiredness) (http://purl.obolibrary.org/obo/HP_0012378)

5. **BULBAR SIGNS**
   - Dysarthria (http://purl.obolibrary.org/obo/HP_0001260)
   - Bulbar signs (http://purl.obolibrary.org/obo/HP_0002483)
   - Dysphagia (http://purl.obolibrary.org/obo/HP_0002015)
   - Palatal myoclonus (http://purl.obolibrary.org/obo/HP_0010530)
   - Vomiting (http://purl.obolibrary.org/obo/HP_0002013)
   - Dysphonia (http://purl.obolibrary.org/obo/HP_0001618)

- Choking episodes (http://purl.obolibrary.org/obo/HP_0030842) (Behavioral abnormality, (http://purl.obolibrary.org/obo/HP_0000708)

6. **OTHER BRAINSTEM SIGNS**
   - Excessive daytime somnolence (http://purl.obolibrary.org/obo/HP_0001262)
   - Sleep apnea (http://purl.obolibrary.org/obo/HP_0010535)
   - Mechanical ventilation (http://purl.obolibrary.org/obo/NCIT_C70909)
   - Central Hypoventilation (http://purl.obolibrary.org/obo/HP_0007110)
   - Dyspnea (http://purl.obolibrary.org/obo/HP_0002094)
   - Hyperventilation (http://purl.obolibrary.org/obo/HP_0002883)
   - (Bilateral) ptosis | (http://purl.obolibrary.org/obo/HP_0001488)
   - Nystagmus (http://purl.obolibrary.org/obo/HP_0000639)

7. **SOMATIC SENSORY DYSFUNCTION & ABNORMALITY OF THE AUTONOMIC NERVOUS SYSTEM**
   - Paresthesia (http://purl.obolibrary.org/obo/HP_0003401)
   - Somatic sensory dysfunction (http://purl.obolibrary.org/obo/HP_0003474)
   - Neurogenic bladder (http://purl.obolibrary.org/obo/HP_0000011)
   - Urinary incontinence (http://purl.obolibrary.org/obo/HP_0000020)
   - Nocturia (http://purl.obolibrary.org/obo/HP_0000017)
   - Impotence (http://purl.obolibrary.org/obo/HP_0000802)
   - Hyperhidrosis (http://purl.obolibrary.org/obo/HP_0000975)
   - Orthostatic hypotension due to autonomic dysfunction (http://purl.obolibrary.org/obo/HP_0004926)

8. **PHENOTYPIC ABNORMALITY** (http://purl.obolibrary.org/obo/HP_0000118)
   - Failure to thrive (http://purl.obolibrary.org/obo/HP_0001508)
   - Weight loss (http://purl.obolibrary.org/obo/HP_0001824)
   - Cachexia (http://purl.obolibrary.org/obo/HP_0004326)
   - Macrocephaly (http://purl.obolibrary.org/obo/HP_0000256)
   - Hydrocephalus (http://purl.obolibrary.org/obo/HP_0000238)
   - Short stature (http://purl.obolibrary.org/obo/HP_0004322)
   - Scoliosis (http://purl.obolibrary.org/obo/HP_0002650)
   - Hip dislocation (http://purl.obolibrary.org/obo/HP_0002827)
   - Hypertelorism (http://purl.obolibrary.org/obo/HP_0000316)
   - Equinus contracture of the ankle (disorder) | http://snomed.info/id/202290007
   - Depressed nasal bridge (http://purl.obolibrary.org/obo/HP_0005280)
   - Precocious puberty (http://purl.obolibrary.org/obo/HP_0000826)
   - Strabismus (http://purl.obolibrary.org/obo/HP_0000486)

MRI abnormalities

A. Supratentorial MRI abnormalities
B. Infratentorial MRI abnormalities
U. Unspecified MRI abnormalities
