# Supplementary material for: A systematic review and meta-analysis of GFAP gene variants in Alexander disease
Source: Sci Rep. 2024 Oct 17;14:24341. doi: 10.1038/s41598-024-75383-4 (PMC11487261; doi:10.1038/s41598-024-75383-4)
Supplement: Supplementary file 3 — Supplementary Material 3 (Figures S1-S4) [file 41598_2024_75383_MOESM3_ESM.pdf]

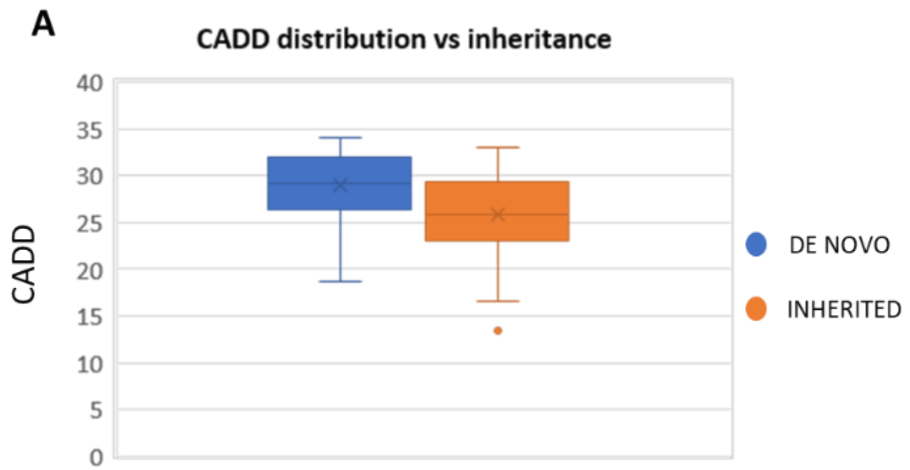

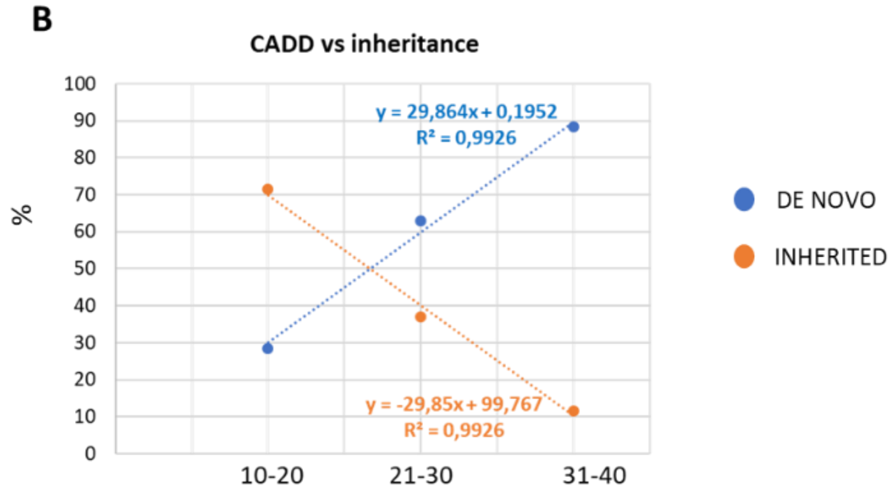

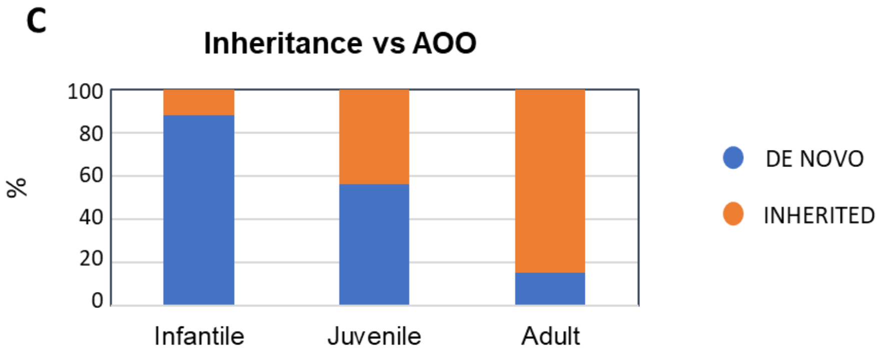

Supplementary Figure S1. Relationship between CADD, inheritance and age of onset

(A) Box graphs represent the CADD distribution between de novo (blue) and inherited (orange) variants.

(B) Percentage of variants de novo (blue dots) and inherited (orange dots) represented in the three CADD intervals 10-20, 21-30, and 31-40 (Variant with CADD 43 is absent because of lack of information on the inheritance). The equations of the regression lines and coefficients of correlation R2 have been calculated.

(C) The graph bar represents the percentage of de novo (blue) and inherited (orange) variants in infantile, juvenile and adult patients.

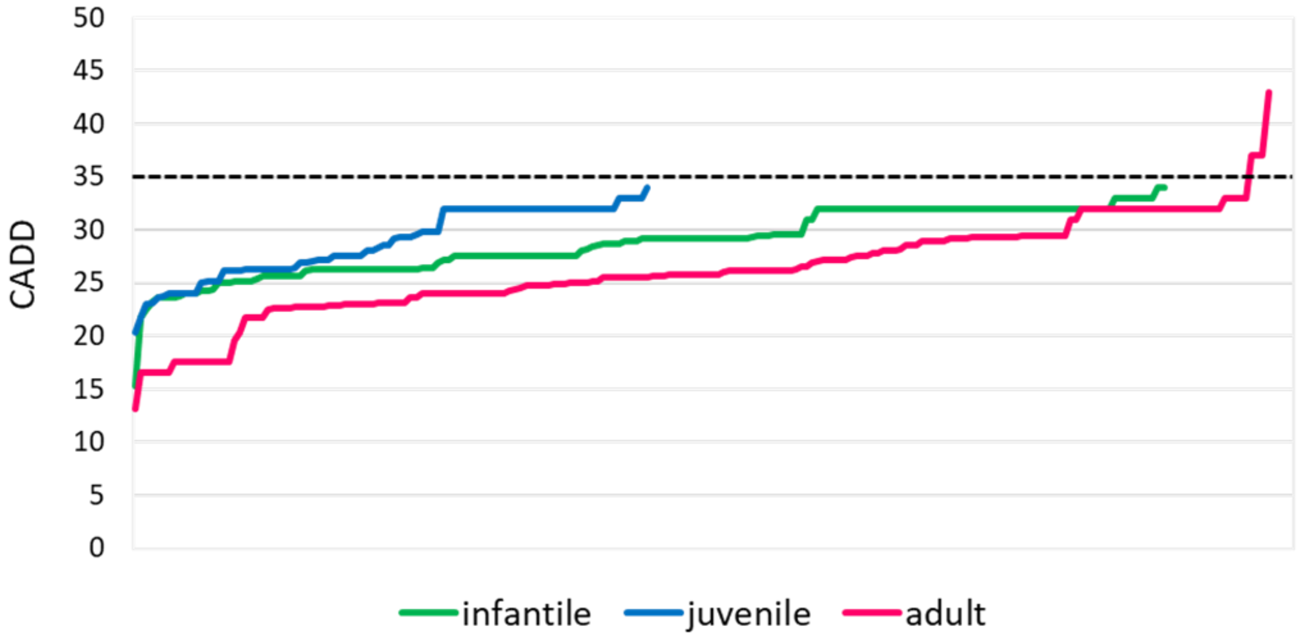

Supplementary Figure S2. CADD distribution in infantile, juvenile and adult patients

Distribution of CADD score along the three AOO groups is shown starting from the lowest to the highest CADD value. The black dashed line indicates the cut-off CADD =35.

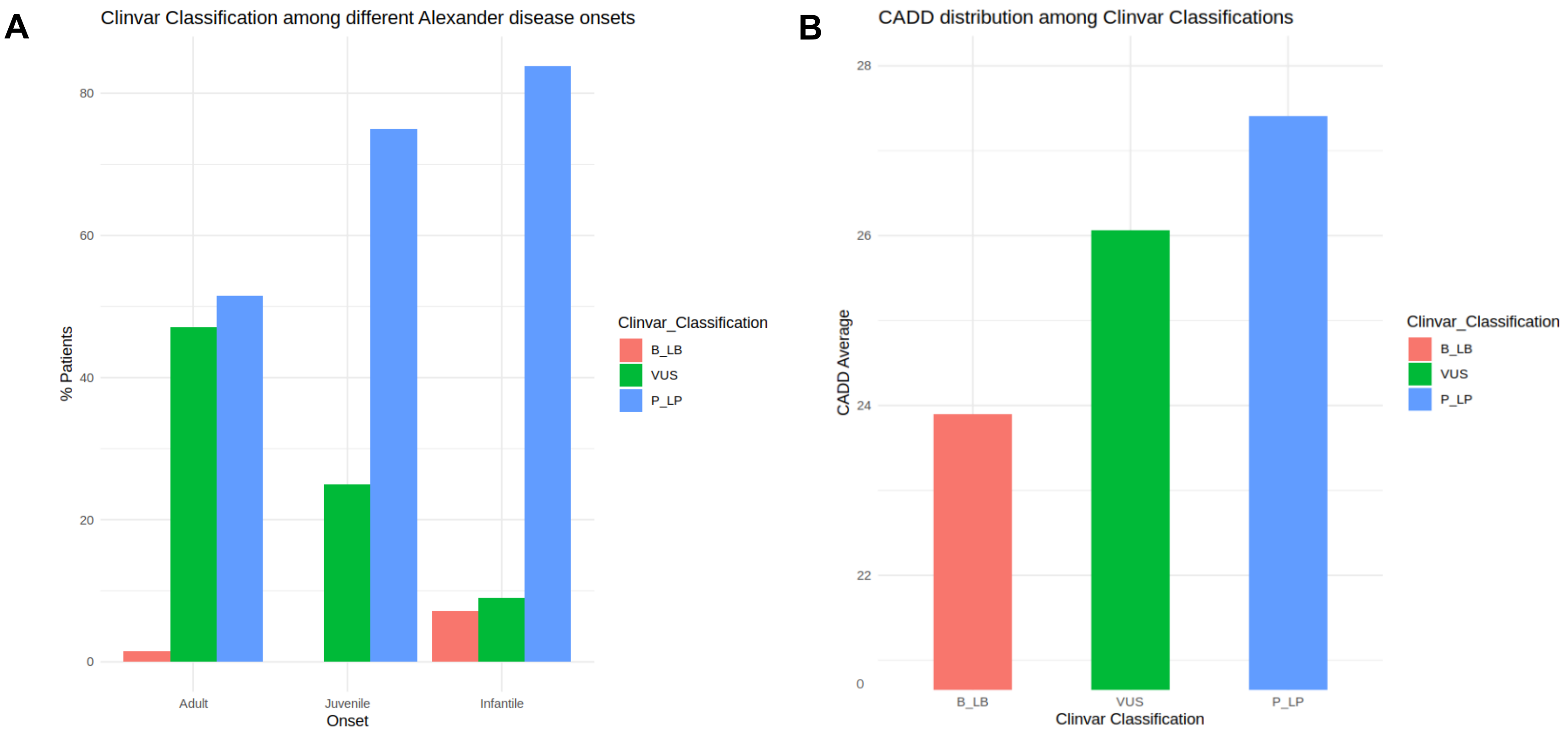

Supplementary Figure S3. ClinVar Classification among ALXDRD patients and correlation with CADD values

(A) Distribution of the ClinVar classification classes in infantile, juvenile and adult patients. With the exception of B/LB that shows a poor correlation (r^2 = 0.567, p-value = 0.4569), VUS (r^2 = 0.992, p-value = 0.05847) and P/LP (r^2 = 0.935, p-value = 0.1641) are correlated with the age of onset, with opposite trends

(B) Average CADD score values correlate with the ClinVar classification classes. As expected, a statistically significant CADD increase is evident from benign to VUS and to pathogenic variants (r2 = 0.329, p-value = 0.01138).

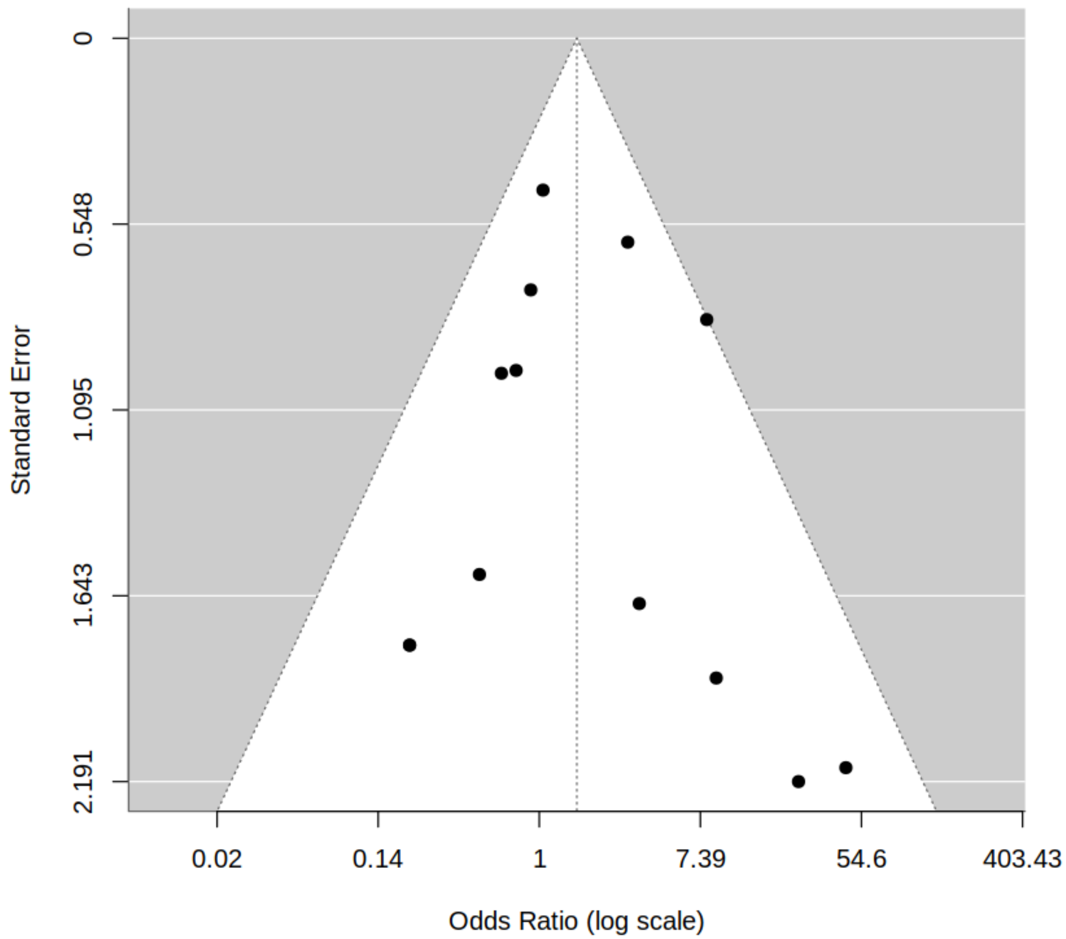

Supplementary Figure S4. Funnel Plot to highlight possible publication bias in Arg vs non-Arg meta-analysis.

Funnel plot of published studies comparing the effects induced by Arg and non-Arg variants. The circles represent a study; the triangle, the region in which 95% of the data would be found in the absence of small study errors; vertical dotted line, odds ratio from meta-analysis. The correlation between study size and intervention effects did not produce any heterogeneity resulting in a symmetric funnel plot.
